# Application of a SODOSM-based MCDM method for evaluating comprehensive fruit quality: A case study of pineapple

**DOI:** 10.1371/journal.pone.0330496

**Published:** 2025-09-02

**Authors:** Ru Zhou, Ling Lin, Qi Li, Shaodong Zeng, Jianzhi Ye, Cheng Luo

**Affiliations:** 1 Agricultural Products Processing Research Institute, Chinese Academy of Tropical Agricultural Sciences, Zhanjiang, Guangdong, China; 2 Laboratory of Quality & Safety Risk Assessment on Agro-products Processing (Zhanjiang), Ministry of Agriculture and Rural Affairs, Zhanjiang, Guangdong, China; Lusofona University of Humanities and Technologies: Universidade Lusofona de Humanidades e Tecnologias, PORTUGAL

## Abstract

This study constructs an evaluation index system and model by integrating objective evaluation methods such as the entropy weight method and TOPSIS with subjective evaluation methods like Delphi and AHP. By analyzed the scatter plots of the comprehensive quality evaluation values of commodity quality before and after weighting, it was found that the quality evaluation values before and after weighting are highly correlated, with correlation coefficients of 0.908 respectively. The weighting method employed in this study only weights the quality evaluation values within a certain range to reflect the subjective preference of consumers, without destructively altering the quality evaluation. This study aims to construct an evaluation index system and model that not only satisfies subjective preferences but also objectively reflects the true quality of pineapples from multiple indicators and dimensions.

## Introduction

Fruits contain essential nutrients, like dietary fiber, minerals, vitamins, and functional substances, which are important components of the human diet [1]. At present, consumers are increasingly concerned about eating quality, nutrition, and health but often lack the knowledge or means to make informed decisions regarding the sustainability of food products beyond food labels such as organic [2] or nutritional characteristics [3,4]. Pineapple represents a crucial tropical fruit crop that occupies a significant position in China’s fruit market. According to the statistical report, China’s pineapple production exceeded 2 million tons [5]. Systematic evaluation of pineapple quality and production systems holds substantial importance for optimizing market supply structures and enhancing overall product quality and competitiveness. Through comprehensive assessment frameworks, stakeholders can accurately quantify production efficiencies and quality parameters, thereby enabling data-driven agricultural decisions that promote premium and green development pathways for this crop.

Modern customers care about fruit quality, nutrition, and health, driving demand for high-grade agricultural products and expectations. Consumers often notice a change in flavor or aroma in agricultural products stored for a long period or marketed in different places, but it is hard to tell the difference. Therefore, it is crucial to evaluate the quality of agricultural products accurately. However, the study of agricultural product quality is still in the research stage, with problems such as unclear quality, confusing evaluation criteria, lacking evaluation methods, and a low research output conversion rate. Providing rational and evidence-based guidance and recommendations for consumers and the industry is difficult.

Scholars from different perspectives have selected various evaluation criteria and methods to construct systems and conduct quality evaluation research on various fruits. However, these studies mostly focus on a single or a few quality characteristics (like flavor, appearance, and nutritional quality) and conduct a single or limited criteria evaluation, lacking a systematic evaluation criteria system for various aspects of quality, such as processing, nutrition, taste, goods, and storage. Hajare conducted a study on the Indian litchi cultivars Shahi and China, analyzing the hardness and brittleness of the fruit peel, vitamin C content, reducing sugars, and flavonoid content [6]. They concluded that these two cultivars are suitable for juice production, but the study did not consider the impact of fruit size and flavor on juice quality. Shan Fu evaluated the effects of soil amendments on pineapple yield, soluble solids, and vitamin C content to assess the effectiveness of different treatment techniques [7]. Although this study considered multiple indicators, each was analyzed independently without a comprehensive evaluation. Bo-Kai Liao investigated the differences in microbial, chemical, and sensory properties of pineapple stored at 5–25°C, but only used total viable counts and weight loss as key indicators to determine the shelf life of pineapple [8].

Multicriteria decision making(MCDM) helps decision-makers to identify the optimal solution from several feasible alternatives based on conflicting quantitative and qualitative criteria, thereby enabling transparent and appropriate decision-making in complex environments [9]. Several review articles [10,11] have summarized and analyzed the application of MCDM methods in different fields, including their advantages, limitations, and future development directions. In MCDM situations, the criteria’ relative relevance varies, hence weighting is needed to represent this [12]. Generally, weights can be obtained through subjective, objective, or a combination of the two [13]. Subjective methods like Analytic Hierarchy Process (AHP), Best-Worst Method (BWM), and Sorting and Weighted Assessment Ratio Analysis (SWARA) rely entirely on decision makers’ subjective judgments. Contrarily, objective methods like Entropy Weight Method and CRITIC method use statistical evaluation of the decision matrix or mathematical models to calculate weights without considering decision makers’ preferences. However, objective methods overlook the importance of human subjective evaluation, which is crucial for making reliable decisions. Contrarily, subjective approaches suffer from subjective inconsistency despite considering human choice. Albahri pointed out that subjective and objective methods fail to consider the importance of standard values, and to address this issue, they introduced the subjective-objective decision-making based on subjective and objective scores method(SODOSM), which combines the strengths of subjective and objective methods to integrate and solve this problem [14].

## Materials and methods

### Test method

According to the requirements of Chinese industry standards GH/T 1154 [15], pineapple samples were randomly selected from the farmers’ market and wholesale market in Zhanjiang City, Guangdong Province. The measurements were conducted as follows: The content of total polyphenols was determined by Folin phenol method, and the standard curve was made with gallic acid as the standard. Total flavonoid content was assessed according to the method described in the Chinese Pharmacopoeia [16]. The peel color L was measured using a colorimeter. Juice yield was calculated as the ratio of the amount of juice extracted to the mass of the fruit. The total sugar content was determined by phenol sulfuric acid method with the aid of spectrophotometer. The titratable acid content in pineapple pulp was determined by sodium hydroxide solution titration. The content of vitamin C in pineapple pulp was determined by 2,6-dichloroindophenol method. Moisture content was determined according to Chinese National Standards GB 5009.3 [17]. Freshness was assessed by visual inspection in accordance with Chinese industry standards NY/T 750 [18]. The mechanical damage fruit ratio was determined by visual inspection, with fruits having a trauma area ≥4% being classified as mechanically damaged. This ratio was calculated as the proportion of mechanically damaged fruits to the total number of samples. The mechanical damage fruit ratio and weight method were used to determine the specification and edible rate. Freshness and peculiar taste were scored according to the criteria outlined in Table 1.

**Table 1:** Freshness and peculiar taste rating requirements.

Freshness	Shiny and full flesh	Dull luster, hard pulp	Dull, pulp collapse
score	8-10	6-8	0-6
peculiar taste	Rich fragrance, no peculiar taste	The fragrance becomes weak, without peculiar smell	No pineapple flavor and peculiar smell
score	0-2	2-4	4-10

### Device

S-433D amino acid analyzer, manufactured by SYKAM GmbH, Germany; GC-2010Plus gas chromatograph (Japan), manufactured by Shimadzu Corporation, Japan; WYA-Z automatic Abbe refractometer, manufactured by Shanghai Precision Scientific Instrument Co., Ltd., China; 722N visible spectrophotometer, manufactured by Shanghai Yidian Scientific Instrument Co., Ltd., China; CR22N high-speed refrigerated centrifuge, manufactured by Hitachi, Ltd., Japan; ANDGX600 electronic balance, manufactured by Shanghai AND Co., Ltd., China; KJELTEC8400 automatic Kjeldahl nitrogen analyzer, manufactured by FOSS, Sweden; CT3-50K texture analyzer, manufactured by Brookfield Engineering Laboratories, Inc., USA; NR20XE precision colorimeter, manufactured by Shenzhen Kerui Technology Co., Ltd., China; FD115 hot air circulation drying oven, manufactured by BINDER GmbH, Germany.

### Quality assurance

All samples were analyzed at the center for food quality testing (Zhanjiang) ministry of agriculture and rural affairs PRC, which holds CMA accreditation. The equipment used in the experiments had been calibrated or functionally verified. Additionally, the personnel involved in sensory evaluation had undergone professional training, and one of their core responsibilities was to conduct sensory assessments of food products, thereby ensuring the reliability and validity of the test results.

### Construction of evaluation model

MCDM approaches generally involve problem definition, alternative solution selection, criteria establishment, decision matrix creation, weight derivation (determination of criterion weights), and alternative solution ranking.

This framework initially develops the decision matrix and produces the standard weights, showing each criterion’s relative relevance in the fruit quality rating index system. Then, the technique for order preference by similarity to ideal solution(TOPSIS) method is used to evaluate and rank the quality of each sample based on the decision matrix. This study employed the SODOSM-based MCDM method, which includes the following steps.

### Subjective weights determination by AHP

MCDM’s computational steps are calculating standard weights and ranking feasible alternative solutions. In the context of MCDM, many methods are available for determining weights, including objective and subjective weight methods.

This study optimizes subjective weights of numerous criteria using AHP-entropy weighting. The AHP method subjectively allocates weights by allowing experts to evaluate the relative importance of criteria [19], thereby reflecting the intentions and preferences of decision-makers. However, the subjective nature of this approach renders it susceptible to biases. To address this, entropy weighting optimizes weights. Information theory uses entropy to quantify uncertainty. When the entropy is small, the corresponding criteria contain more information and therefore carry greater weight [20], reflecting the characteristics of the data. Neglecting subjective opinions may result in missed decision-maker intentions and preferences. Thus, AHP-entropy weighing combines subjective and objective methodologies to maximize their strengths and minimize their limitations. The specific steps involved in the relevant calculations refer to Miguel Ortiz Barios’ research [21].

### Objective weights determination by entropy

The entropy weight method is capable of automatically calculating the weights of various indicators through objective data analysis, thereby avoiding biases caused by human interference. By incorporating the concept of entropy from information theory, entropy weight method can effectively measure the importance of each indicator in decision-making.

In the entropy weight method, positive indicators are defined as those for which higher values are preferable, while negative indicators are those for which lower values are desirable. Interval indicators, on the other hand, exhibit optimal performance within a specific numerical range. To standardize the processing of these distinct indicator types, it is typically necessary to apply positive-oriented transformation techniques to negative and interval indicators, thereby converting them into equivalent positive indicators.The specific steps involved in the relevant calculations refer to Yuxin Zhu’s research research [22].

Interval-type indicators refer to those for which the optimal value falls within a specific fixed range, or in other words, the closer the value is to a certain fixed range (including falling within that range), the better. In this study, the acid-sugar ratio was selected as an important indicator for evaluating the sensory quality of fruits. This ratio reflects the balance between sweetness and acidity in fruits, directly influencing the taste experience. For fresh fruits, a higher SAR indicates a predominantly sweet flavor, while a lower acid-sugar ratio suggests a more acidic taste. For most people, fruits with an acid-sugar ratio between 8 and 20 are considered to have a well-balanced sweet and sour taste. When the acid-sugar ratio exceeds 20, only a very slight sourness can be detected, whereas an acid-sugar ratio below 5 results in a significantly sour taste.

### Comprehensive weights

The comprehensive weight w of the second-level criteria was computed using the following method:


$${\text{w}}_{\text{j}}=\frac{\sqrt{\alpha_{\text{j}}\beta_{\text{j}}}}{\sum_{\text{j}=\text{1}}^{\text{n}}\sqrt{\alpha_{\text{j}}\beta_{\text{j}}}}$$ (1)

In the equation, the symbol β_j_ denotes the objective weights; the symbol ɑ_j_ denotes the subjective weights.

#### Sample scoring values determination by TOPSIS.

TOPSIS assumes that the optimal solution has the smallest Euclidean distance to the positive ideal solution and the maximum to the negative ideal solution [20]. The TOPSIS method provides trade-offs between criteria. Thus, all criteria contribute to the optimum answer, and one criterion’s weakness can be offset by another’s strength. However, the TOPSIS method has certain limitations in that it requires explicit knowledge of the weights of the known criteria, which should be considered in the method. In this study, we propose integrating the SODOSM method into the TOPSIS framework to address the limitations of existing evaluation approaches. By assigning comprehensive weights to each indicator through a combination of subjective and objective methods, the evaluation of each sample is adjusted to conform to both subjective consumption tendencies and objective quality attributes. This integrated approach ensures a more balanced and holistic assessment, thereby enhancing the robustness and applicability of the evaluation results. The specific steps involved in the relevant calculations refer to Miguel Ortiz Barios’ research [21].

### Statistical data analysis

WPS(KINGSOFT OFFICE, Beijing,China) were used to analyze the obtained results。Matrix eigenvalues are calculated using yunsuanwang (http://www.yunsuan.info/).

## Results and discussion

### Criteria for the evaluation of quality

Scientific assessment principles are essential for efficient instructional evaluation. To reflect the scientific nature of evaluation to the greatest extent, evaluation criteria should be designed to reflect the characteristics of the evaluated object from multiple perspectives and levels around the evaluation objectives and ensure that evaluation criteria are non-redundant and easily observable.

Scientific and comprehensive product quality evaluation is essential for agricultural development and consumer protection. To achieve this goal, it is essential to establish a relatively complete set of evaluation criteria and methods that fully reflect the quality characteristics of agricultural products and consider the needs and tendencies of consumers and industries, making it research-oriented and applicable. Decision-making criteria can be divided into three types: maximal standards (the higher the score, the better the performance, like profit), minimal standards (the lower the score, the better the performance, like price) [11,23], and intermediate and interval criteria.

After reviewing the CAC standards, Chinese agricultural industry standards, relevant literature, and conducting on-site investigations of fruit cultivation, transportation, processing enterprises, and consumers, we developed an MCDM evaluation index system comprising 15 secondary evaluation criteria that cover four primary criteria. Secondary criteria are chosen based on eating, processing, nutritional, and commodity qualities.

The eating quality assessment includes four factors: freshness, off-flavor, sugar-acid ratio, and the L value of fruit peel color. Titratable acidity, juice production, and moisture content determine processing quality. Nutritional quality assessment encompasses five factors: polyphenols, flavonoids, total sugar, vitamin C, and soluble solids. Commodity quality evaluation involves single fruit weight, edible rate, and mechanical damage. The MCDM evaluation index system is presented in S1 Table.

In this study, we obtained 23 pineapple samples from China’s primary pineapple-producing regions, including Guangdong, Guangxi, Hainan, and Fujian, to demonstrate our research technique. The detection data of 23 pineapple samples are shown in S2 Table.

### Subjective weights of pineapple

The AHP 1–9 value technique assessed the relative relevance of first- and second-level criteria, and the judgment matrix calculated their weights. After verification, CR ≤ 0.1, and the consistency test was passed, which conforms to the principle of AHP. The results are shown in Table 2.

**Table 2:** Relative importance between criteria.

Relative importance between first-level criteria							
	eating quality			processing quality	nutritional quality	commodity quality	
eating quality	1			9	5	3	
processing quality	1/9			1	1/3	1/7	
nutritional quality	1/5			3	1	1/4	
commodity quality	1/3			7	4	1	
Relative importance between second-level criteria within the eating quality criteria							
	fresh degree			peculiar taste	Peel color L		acid-sugar ratio
fresh degree	1			1/5	3		2
peculiar taste	5			1	8		6
Peel color L	1/3			1/8	1		1/2
acid-sugar ratio	1/2			1/6	2		1
Relative importance between second-level criteria within the processing quality criteria							
	Titratable acid			rate of juice extracting			moisture
Titratable acid	1			1/4			1/2
rate of juice extracting	4			1			3
moisture	2			1/3			1
Relative importance between second-level criteria within the nutritional quality criteria							
	polyphenol	flavone	total sugar		Vitamin C		soluble solid
polyphenol	1	4	4		1/4		3
flavone	1/4	1	1		1/6		1/2
total sugar	1/4	1	1		1/6		1/2
Vitamin C	4	6	6		1		5
soluble solid	1/3	2	2		1/5		1
Relative importance between second-level criteria within the commodity quality criteria							
	physical injury			single fruit weight			edible rate
physical injury	1			3			5
single fruit weight	1/3			1			2
edible rate	1/5			1/2			1

The Table 3 below shows the pineapple quality evaluation index system’s first- and second-level criteria weights. Taste quality has the greatest impact on pineapple quality, followed by commodity and nutritional quality. Processing quality has the least impact because pineapple is mostly consumed fresh and processed as a supplement. Off-flavor gives customers an unpleasant sensory experience and often impacts their intake propensity. In this study, the weight of off-flavor is the largest, consistent with actual consumption tendencies.

**Table 3:** Indicator subjective weight.

Goal	first-level criteria	subjective contribution weight α_t_	second-level criteria	subjective contribution weights α_tj_	subjective weights α_j_
Comprehensive Quality Evaluation of Pineapple	eating quality	0.5658	fresh degree	0.174	0.0984
			peculiar taste	0.6572	0.3718
			Peel color L	0.0631	0.0357
			acid-sugar ratio	0.1056	0.0597
	processing quality	0.0441	Titratable acid	0.1365	0.006
			rate of juice extracting	0.625	0.0276
			moisture	0.2385	0.0105
	nutritional quality	0.1015	polyphenol	0.2316	0.0235
			flavone	0.0636	0.0065
			total sugar	0.0636	0.0065
			Vitamin C	0.5353	0.0543
			soluble solid	0.1058	0.0107
	commodity quality	0.2887	physical injury	0.6483	0.1872
			single fruit weight	0.2297	0.0663
			edible rate	0.122	0.0352

### Objective weights of pineapple

Multiple factors are weighted objectively using the entropy weight approach. The primary idea is to weigh the criteria using the difference between information objectively. The evaluation results mainly depend on the discreteness of the data itself; thus, it is not easily affected by human factors. The objective weights of second-level criteria are calculated from 23 standardized values after sample conversion, as shown in Table 4. From the table, criteria with large differences in values like mechanical damage, off-flavor, and single fruit weight have larger weights and can better reflect differences between samples.

**Table 4:** Criteria Weight Allocation Table.

second-level criteria	The entropy value (H_j_)	Objective weights (β_j_)
fresh degree	0.9967	0.0136
peculiar taste	0.9551	0.1845
Peel color L	0.9941	0.0242
acid-sugar ratio	0.9593	0.1673
Titratable acid	0.9961	0.016
rate of juice extracting	0.9992	0.0033
moisture	0.9998	0.0008
polyphenol	0.9891	0.0448
flavone	0.9967	0.0136
total sugar	0.9946	0.0222
Vitamin C	0.9822	0.0732
soluble solid	0.996	0.0164
physical injury	0.9228	0.3173
single fruit weight	0.9756	0.1003
edible rate	0.9994	0.0025

### Comprehensive weights of pineapple

**Fig 1:**
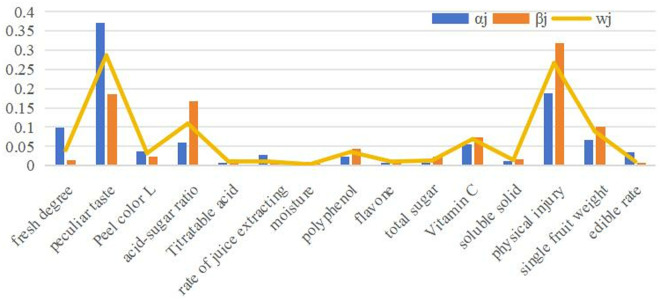
Weights of criteria. (α_j_) subjective weights; (β_j_) objective weights; (wj)Comprehensive weights.

From Fig 1, humans subjectively prefer to choose taste quality as the standard for selecting pineapples. However, pineapple quality is primarily a matter of commodity. Therefore, if subjective or objective evaluation methods are used alone, one is that human subjective consciousness intervenes too much, which is easy to ignore the unique quality of pineapples, resulting in the evaluation index system not matching the actual application; two is that the existing index weight assignment method is too objective and lacks subjective initiative, and consumer subjective preferences are not reflected. Consumers may dislike the best evaluation. Thus, utilizing entropy weight to change index weight ensures objectivity and fairness of evaluation findings.

### Sample scoring values of pineapple

**Fig 2:**
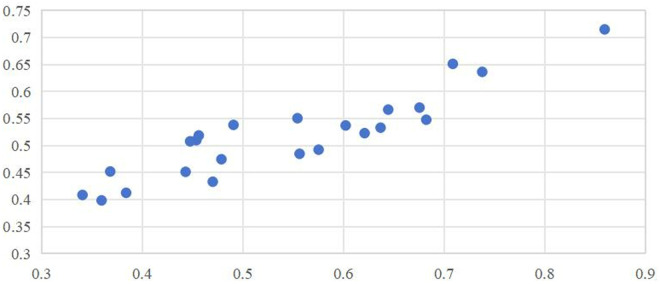
Scatter plot of sample quality scores.

The comprehensive quality evaluation scores and rankings of the 23 samples before and after weighting are shown in Table 5. The table shows that weighted lychee thorough quality evaluation has an effect, specifically on samples with special quality in one area. As shown in Figure 2, by analyzing the scatter plots of the comprehensive quality evaluation values of comprehensive, taste, processing, nutritional, and commodity quality before and after weighting, it is found that the quality evaluation values before and after weighting are highly correlated, with correlation coefficients of 0.873, 0.900, 0.998, 0.942 and 0.950 respectively. This study’s weighing mechanism is also scientifically feasible. It only weights the quality evaluation values to a certain extent to reflect consumers’ subjective consciousness preferences and does not cause destructive changes to the quality evaluation.

**Table 5:** Comprehensive Quality Score of Pineapple Samples Before and After Weighting.

Sample number	Comprehensive Quality score		sorting	
	After weighting	Before weighting	After weighting	Before weighting
1	0.8598	0.7152	1	1
2	0.4433	0.4514	19	19
3	0.4703	0.4331	15	20
4	0.6825	0.5479	4	7
5	0.6758	0.5703	5	4
6	0.5545	0.5507	12	6
7	0.7381	0.6365	2	3
8	0.6212	0.523	8	11
9	0.4909	0.5383	13	8
10	0.4789	0.4747	14	17
11	0.4478	0.5079	18	14
12	0.4564	0.5188	16	12
13	0.7089	0.6513	3	2
14	0.3684	0.452	21	18
15	0.3842	0.4125	20	21
16	0.6371	0.5332	7	10
17	0.454	0.51	17	13
18	0.3408	0.4086	23	22
19	0.5566	0.4848	11	16
20	0.3598	0.3985	22	23
21	0.6024	0.5373	9	9
22	0.5755	0.4924	10	15
23	0.6447	0.5666	6	5

## Discussion

China maintains global leadership in both fruit production and consumption, with annual output reaching 320 million tons in 2023 – the highest worldwide for seven consecutive years. The per capita consumption exceeds 65 kg/year, surpassing WHO recommendations. However, challenges persist regarding product homogeneity and post-harvest surplus. Recent national initiatives including the “Three Products and One Standard” agricultural campaign (encompassing cultivar improvement, quality enhancement, brand development, and standardized production) aim to optimize industrial ecology through quadrilateral coordination [24].

This study contributes to advancing quality evaluation systems and premium pricing mechanisms, thereby facilitating high-quality development in the fruit industry. Through our research, we have established a quality evaluation system primarily consisting of 15 indicators such as sugar-acid ratio, vitamin C, and single fruit weight. Using two mathematical statistical analysis methods, namely Delphi and AHP, we calculated the comprehensive weights of each indicator, which are applied to guide consumption as well as variety breeding and selection. Our analytical hierarchy process (AHP) analysis revealed that physical damage, off-flavor, and freshness received the highest subjective weights (0.216, 0.195, and 0.183 respectively) in pineapple evaluation, emphasizing consumer prioritization of these attributes during purchasing decisions. These findings suggest operational improvements in transportation protocols and storage conditions to minimize mechanical injury and quality deterioration.In terms of objective quality indicators, the weight of acid-sugar ratio、vitamin C and single fruit weight is greater than 0.05, providing targeted direction for cultivar selection. Practical applications include strategic marketing of optimal specimens to ensure marketability, and selective cultivation of premium varieties to establish quality-oriented branding. These evidence-based recommendations offer scientific support for sustainable industry development through integrated quality management and policy optimization.

However, the current established model has many indicators, and the process of determining sample quality evaluation indicators is complex, which is not conducive to the application of management departments in practice. Next, we will expand the sampling area, increase the data volume, and carefully summarize and propose several important indicators using statistical methods such as significant difference tests, and then use the model for calculation and analysis. Meanwhile, future model iterations will integrate economic metrics to align quality assessments with premium pricing mechanisms, thereby reinforcing the “quality-value congruence” framework. Furthermore, we propose developing a computerized evaluation system to automate sample prioritization through algorithmic data processing, eliminating manual computations and significantly enhancing implementation efficiency. This computational tool will streamline quality-driven decision-making while ensuring reproducibility across agricultural applications.

## Conclusion

This study used the MCDM model to construct a comprehensive pineapple quality evaluation index system and model, evaluated the contribution of taste, processing, nutritional, commodity quality, and other qualities to pineapple quality, and evaluated pineapple quality from multiple perspectives. The evaluation index system includes 4 aspects: sensory quality, processability, nutritional value, and commodity quality. Among them, eating quality consists of 4 indicators: fresh degree, peculiar taste, Peel color L, and acid-sugar ratio; processing quality includes 3 indicators: Titratable acid, rate of juice extracting, and moisture; nutritional quality comprises 5 indicators: polyphenol, flavone, total sugar, Vitamin C, and soluble solid; commodity quality contains 3 indicators: physical injury, single fruit weight, and edible rate. Through calculations using the Delphi method and AHP, the weights of the 15 indicators are 0.04, 0.2864, 0.0321, 0.1092, 0.0107, 0.0104, 0.0032, 0.0354, 0.0103, 0.0131, 0.0689, 0.0144, 0.2665, 0.0891, and 0.0103 respectively. On the basis of the evaluation index system, the model of this study is constructed by supplementing the calculation of original data with TOPSIS. Case validation substantiated the model’s practicality and operational feasibility in comprehensive pineapple quality evaluation. The demonstrated generalizability through sample substitution suggests broader applicability to diverse fruit varieties.

## Supporting information

S1 Table The MCDM evaluation index system. (DOCX)

S2 Table The detection data of 23 pineapple samples. (DOCX)

S1 File The values used to build Figure 1. (DOCX)

S2 File The values used to build Figure 2. (DOCX)
