# Supplementary material for: Application of a SODOSM-based MCDM method for evaluating comprehensive fruit quality: A case study of pineapple
Source: PLoS One. 2025 Sep 2;20(9):e0330496. doi: 10.1371/journal.pone.0330496 (PMC12404366; doi:10.1371/journal.pone.0330496)
Supplement: S1 Table — (DOCX) [file pone.0330496.s001.docx]

**S1 Table The MCDM evaluation index system**

| Goal | first-level criteria | second-level criteria | The meaning of the criteria | criteria type |
| --- | --- | --- | --- | --- |
| Comprehensive Quality Evaluation of Pineapple | eating quality(B_1_) | fresh degree(C_1_) | The freshness of the fruit. | maximization-type |
|  |  | peculiar taste(C_2_) | The abnormal odor or taste of fruit results from spoilage or absorption of undesirable odors. | minimization-type |
|  |  | Peel color L(C_3_) | The brightness of the fruit peel increases with the index value, meaning that the higher the index value, the brighter the fruit peel. | maximization-type |
|  |  | acid-sugar ratio(C_4_) | The proportional relationship between total sugar content and titratable acid content. | interval-type |
|  | processing quality(B_2_) | Titratable acid(C_5_) | Titratable acidity is one of the important constituent traits of plant quality and a crucial factor affecting the flavor quality of fruits. | maximization-type |
|  |  | rate of juice extracting(C_6_) | The percentage of fruit quality to the juice extracted from it. | maximization-type |
|  |  | moisture(C_7_) | The weight loss of the sample upon constant drying at 100-105℃. | maximization-type |
|  | nutritional quality(B_3_) | polyphenol(C_8_) | Polyphenols are secondary metabolites with diverse polyphenolic structures widely distributed in plant organisms and are compounds with potential health-promoting effects. | maximization-type |
|  |  | flavone(C_9_) | Flavonoids refer to a series of compounds that contain two phenolic hydroxyl groups on two benzene rings (A- and B-rings) connected by a central three-carbon atom with a basic nucleus of 2-phenylchromenone. | maximization-type |
|  |  | total sugar(C_10_) | Total sugar is the sum of all the sugars that can be absorbed, digested, and utilized by the human body. | maximization-type |
|  |  | Vitamin C(C_11_) | Vitamin C is a polyhydroxy compound with the chemical formula C_6_H_8_O_6_. | maximization-type |
|  |  | soluble solid(C_12_) | The term for the collective presence of water-soluble sugars, acids, proteins, vitamins, pectin, and other compounds in fruits. | maximization-type |
|  | commodity quality(B_4_) | physical injury(C_13_) | Injuries caused by mechanical forces. | minimization-type |
|  |  | single fruit weight(C_14_) | Indicating the size of the fruit. | maximization-type |
|  |  | edible rate(C_15_) | The edible rate refers to the ratio of the edible portion to the weight of the whole fruit. | maximization-type |
