# Supplementary material for: Application of a SODOSM-based MCDM method for evaluating comprehensive fruit quality: A case study of pineapple
Source: PLoS One. 2025 Sep 2;20(9):e0330496. doi: 10.1371/journal.pone.0330496 (PMC12404366; doi:10.1371/journal.pone.0330496)
Supplement: S2 Table — (DOCX) [file pone.0330496.s002.docx]

**S2 Table The detection data of 23 pineapple samples**

| Sample number | eating quality | | | | processing quality | | | nutritional quality | | | | | commodity quality | | |
| --- | --- | --- | --- | --- | --- | --- | --- | --- | --- | --- | --- | --- | --- | --- | --- |
|  | fresh degree | peculiar taste | Peel color L | acid-sugar ratio | Titratable acid | rate of juice extracting | moisture | polyphenol | flavone | total sugar | Vitamin C | soluble solid | physical injury | single fruit weight | edible rate |
| 1 | 8.5 | 0.5 | 44.64 | 10.37 | 6.83 | 72.13 | 87.12 | 70.83 | 82.53 | 70.83 | 31.69 | 20.49 | 0 | 1.99 | 68.32 |
| 2 | 6 | 2.5 | 27.85 | 22.12 | 4.27 | 73.59 | 86.3 | 94.46 | 72.03 | 94.45 | 20.8 | 24.73 | 30 | 1.43 | 72.89 |
| 3 | 7.5 | 1.5 | 41.51 | 24.56 | 4.27 | 68.63 | 79.25 | 104.85 | 62.3 | 104.87 | 15.89 | 23.5 | 30 | 1.21 | 72.76 |
| 4 | 6 | 1 | 36.41 | 21.41 | 5.03 | 62.84 | 82.78 | 107.71 | 46.98 | 107.69 | 13.96 | 18.04 | 10 | 1.13 | 78.88 |
| 5 | 8 | 2 | 44.9 | 17.08 | 5.53 | 75.69 | 81.69 | 114.46 | 54.26 | 94.47 | 11.16 | 19.73 | 0 | 0.83 | 83.44 |
| 6 | 7.5 | 1 | 42.33 | 21.8 | 5.5 | 73.77 | 86.43 | 119.9 | 50.16 | 119.9 | 24.5 | 22 | 30 | 1.39 | 75.56 |
| 7 | 8 | 0.5 | 36.23 | 23.29 | 5.4 | 71.56 | 81.52 | 143.75 | 60.32 | 125.75 | 25.82 | 15.01 | 10 | 1.51 | 67.58 |
| 8 | 6 | 0.5 | 30.26 | 26.73 | 5.35 | 73.48 | 84.74 | 196.51 | 54.88 | 143.01 | 12.91 | 19.16 | 10 | 1.06 | 79.56 |
| 9 | 9 | 3 | 47.58 | 23.01 | 5.81 | 75.35 | 86.22 | 153.67 | 62.25 | 133.67 | 20.27 | 22.13 | 20 | 1.28 | 84.92 |
| 10 | 6.5 | 0.5 | 25.76 | 25.59 | 5.24 | 64.67 | 84.31 | 134.09 | 64.36 | 134.09 | 28.85 | 20.94 | 40 | 1.43 | 81.33 |
| 11 | 7 | 4 | 52.34 | 25.12 | 4.33 | 81.82 | 89.44 | 108.77 | 54.44 | 108.76 | 27.94 | 22.35 | 20 | 2.22 | 67.65 |
| 12 | 6 | 4.5 | 30.94 | 18.77 | 5.92 | 72.45 | 91.1 | 111.12 | 59.93 | 111.11 | 30.29 | 17.6 | 20 | 1.65 | 71.79 |
| 13 | 8 | 2.5 | 35.82 | 15.03 | 6.27 | 76.93 | 79.98 | 166.84 | 67.84 | 94.23 | 23.82 | 14.82 | 0 | 1.38 | 67.03 |
| 14 | 8 | 4 | 29.25 | 16.17 | 6.32 | 77.48 | 91.29 | 195.41 | 59.62 | 102.21 | 27.36 | 17.26 | 30 | 0.38 | 75.75 |
| 15 | 6 | 5 | 34.46 | 21.85 | 3.58 | 64.62 | 81.34 | 78.22 | 62.77 | 78.22 | 35.46 | 17.68 | 20 | 1 | 73.35 |
| 16 | 7 | 1.5 | 31.38 | 20.46 | 6.1 | 82.51 | 83.21 | 144.81 | 54.49 | 124.81 | 11.52 | 13.9 | 10 | 0.64 | 74.4 |
| 17 | 9.5 | 3 | 42.52 | 16.17 | 5.22 | 68.38 | 82.74 | 134.42 | 59.86 | 84.41 | 34.61 | 17.68 | 30 | 0.97 | 76.26 |
| 18 | 6.5 | 3 | 27.72 | 24.23 | 4.8 | 64.2 | 84.86 | 116.3 | 69.28 | 116.3 | 25.28 | 21.94 | 40 | 1.64 | 76.65 |
| 19 | 8.5 | 3 | 35.36 | 18.46 | 3.86 | 68.54 | 87.91 | 71.26 | 75.74 | 71.25 | 24.34 | 17.34 | 10 | 0.51 | 79.06 |
| 20 | 8 | 2.5 | 31.88 | 21.95 | 4.59 | 78.33 | 79.46 | 146.65 | 82.69 | 100.75 | 19.95 | 17.29 | 40 | 0.67 | 68.16 |
| 21 | 7 | 2.5 | 38.91 | 19.33 | 5.11 | 72.41 | 85.35 | 133.78 | 72.34 | 98.77 | 15.85 | 14.71 | 10 | 0.84 | 80.91 |
| 22 | 8.5 | 1 | 34.58 | 22.28 | 4.72 | 75.23 | 89.48 | 152.31 | 68.38 | 105.14 | 11.89 | 15.44 | 20 | 0.66 | 81.71 |
| 23 | 7.5 | 2.5 | 46.67 | 23.97 | 4.83 | 70.55 | 83.26 | 115.73 | 71.29 | 115.78 | 16.97 | 20.35 | 0 | 1.3 | 80.86 |
