# Supplementary material for: Application of a SODOSM-based MCDM method for evaluating comprehensive fruit quality: A case study of pineapple
Source: PLoS One. 2025 Sep 2;20(9):e0330496. doi: 10.1371/journal.pone.0330496 (PMC12404366; doi:10.1371/journal.pone.0330496)
Supplement: S1 File — (DOCX) [file pone.0330496.s003.docx]

**S1 File The values used to build Figure 1**

|  | α_j_ | β_j_ | w_j_ |
| --- | --- | --- | --- |
| fresh degree | 0.0984 | 0.0136 | 0.04 |
| peculiar taste | 0.3718 | 0.1845 | 0.2864 |
| Peel color L | 0.0357 | 0.0242 | 0.0321 |
| acid-sugar ratio | 0.0597 | 0.1673 | 0.1092 |
| Titratable acid | 0.006 | 0.016 | 0.0107 |
| rate of juice extracting | 0.0276 | 0.0033 | 0.0104 |
| moisture | 0.0105 | 0.0008 | 0.0032 |
| polyphenol | 0.0235 | 0.0448 | 0.0354 |
| flavone | 0.0065 | 0.0136 | 0.0103 |
| total sugar | 0.0065 | 0.0222 | 0.0131 |
| Vitamin C | 0.0543 | 0.0732 | 0.0689 |
| soluble solid | 0.0107 | 0.0164 | 0.0144 |
| physical injury | 0.1872 | 0.3173 | 0.2665 |
| single fruit weight | 0.0663 | 0.1003 | 0.0891 |
| edible rate | 0.0352 | 0.0025 | 0.0103 |
