# Supplementary material for: Application of a SODOSM-based MCDM method for evaluating comprehensive fruit quality: A case study of pineapple
Source: PLoS One. 2025 Sep 2;20(9):e0330496. doi: 10.1371/journal.pone.0330496 (PMC12404366; doi:10.1371/journal.pone.0330496)
Supplement: S2 File — (DOCX) [file pone.0330496.s004.docx]

**S2 File The values used to build Figure 2**

| Sample number | After weighting | Before weighting |
| --- | --- | --- |
| 1 | 0.8598 | 0.7152 |
| 2 | 0.4433 | 0.4514 |
| 3 | 0.4703 | 0.4331 |
| 4 | 0.6825 | 0.5479 |
| 5 | 0.6758 | 0.5703 |
| 6 | 0.5545 | 0.5507 |
| 7 | 0.7381 | 0.6365 |
| 8 | 0.6212 | 0.523 |
| 9 | 0.4909 | 0.5383 |
| 10 | 0.4789 | 0.4747 |
| 11 | 0.4478 | 0.5079 |
| 12 | 0.4564 | 0.5188 |
| 13 | 0.7089 | 0.6513 |
| 14 | 0.3684 | 0.452 |
| 15 | 0.3842 | 0.4125 |
| 16 | 0.6371 | 0.5332 |
| 17 | 0.454 | 0.51 |
| 18 | 0.3408 | 0.4086 |
| 19 | 0.5566 | 0.4848 |
| 20 | 0.3598 | 0.3985 |
| 21 | 0.6024 | 0.5373 |
| 22 | 0.5755 | 0.4924 |
| 23 | 0.6447 | 0.5666 |
